# BAT2: an Open-Source Tool for Flexible, Automated, and Low Cost Absolute Binding Free Energy Calculations

**DOI:** 10.1021/acs.jctc.4c00205

**Published:** 2024-08-01

**Authors:** Germano Heinzelmann, David J. Huggins, Michael K. Gilson

**Affiliations:** † Departamento de Fisica, Universidade Federal de Santa Catarina, Florianopolis 88040-970, Brasil; ‡ Department of Physiology and Biophysics, Weill Cornell Medical College of Cornell University, New York, New York 10065, United States; § Sanders Tri-Institutional Therapeutics Discovery Institute, 1230 York Avenue, Box 122, New York, New York 10065, United States; ∥ Skaggs School of Pharmacy and Pharmaceutical Sciences, University of California, San Diego 92093, United States

## Abstract

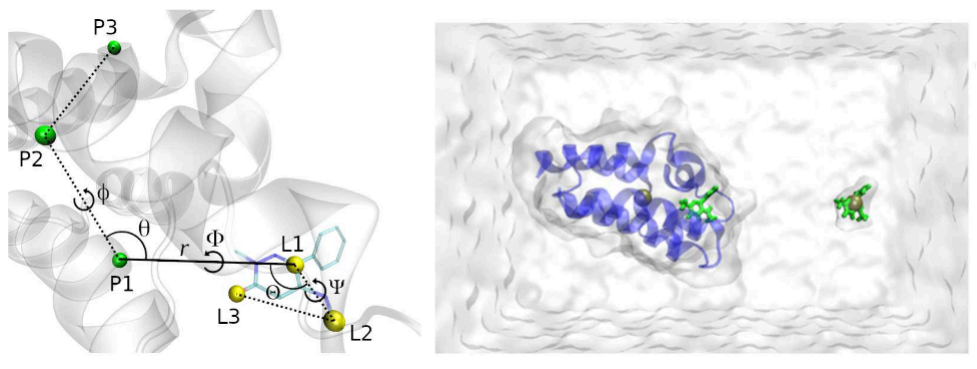

Absolute binding free energy (ABFE) calculations with all-atom molecular dynamics (MD) have the potential to greatly reduce costs in the first stages of drug discovery. Here, we introduce BAT2, the new version of the Binding Affinity Tool (BAT.py), designed to combine full automation of ABFE calculations with high-performance MD simulations, making it a potential tool for virtual screening. We describe and test several changes and new features that were incorporated into the code, such as relative restraints between the protein and the ligand instead of using fixed dummy atoms, support for the OpenMM simulation engine, a merged approach to the application/release of restraints, support for cobinders and proteins with multiple chains, and many others. We also reduced the simulation times for each ABFE calculation, assessing the effect on the expected robustness and accuracy of the calculations.

## 1 Introduction

Identifying molecules that bind to a therapeutic target is one of the most important steps in the early stages of drug discovery. Experimental high-throughput screening (HTS) using robots can be carried out on hundreds of thousands of ligands in a single day,^1,2^ but the need for large compound libraries and sophisticated machinery makes this approach costly and resource intensive. In the past years, virtual screening (VS) using computational scoring functions have emerged as a viable alternative, with many studies identifying potent molecules that were subsequently validated by experiments.^3,4^ Ligand-based VS methods^5,6^ rely on available experimental data, and so do advanced structure-based docking^7,8^ and ranking methods that use artificial intelligence (AI).^9,10^ Consequently, VS on novel targets without existing chemical matter is expected to produce less accurate results.

Another important class of computational tools that can estimate protein–ligand affinities are the physics-based methods, which perform binding free energy calculations using an ensemble of states generated by all-atom molecular dynamics (MD) simulations.^3,11−15^ The interactions between the atoms in the MD simulation are described by an atom-based force-field, so in principle there is a high level of transferability between different biological systems. These free energy methods are commonly divided in two classes, relative (RBFE)^16−20^ and absolute (ABFE)^11,13,21−29^ binding free energy calculations. RBFE methods, as the name suggests, compute the relative difference in binding free energy between two similar compounds, by alchemically transforming one into the other in the receptor binding site, and doing the opposite transformation in bulk solvent. Even though widely used in the lead optimization stages, RBFE calculations can become challenging when comparing molecules that have little similarity,^30^ making it unsuitable for virtual screening on a diverse set of ligands.

Conversely, ABFE calculations estimate the standard binding free energy of a single ligand by calculating the free energy difference of transferring it from the protein binding site to bulk solvent at 1 M concentration. Since each ligand is treated individually and their binding free energies can be directly compared, ABFE can rank ligands regardless of similarity and thus can be applied to virtual screening.^3,13,15^ A recent study by Feng et al. has shown that, despite not yet having the same accuracy as RBFE methods, ABFE calculations can effectively distinguish between binders and nonbinders to a given receptor and further enrich a set of top-scoring compounds obtained by docking.^15^

For many years after the implementation of ABFE calculations, there were a few practical challenges that prevented its widespread adoption. In recent years, these challenges have been partially or completely addressed.

**Figure 1:**
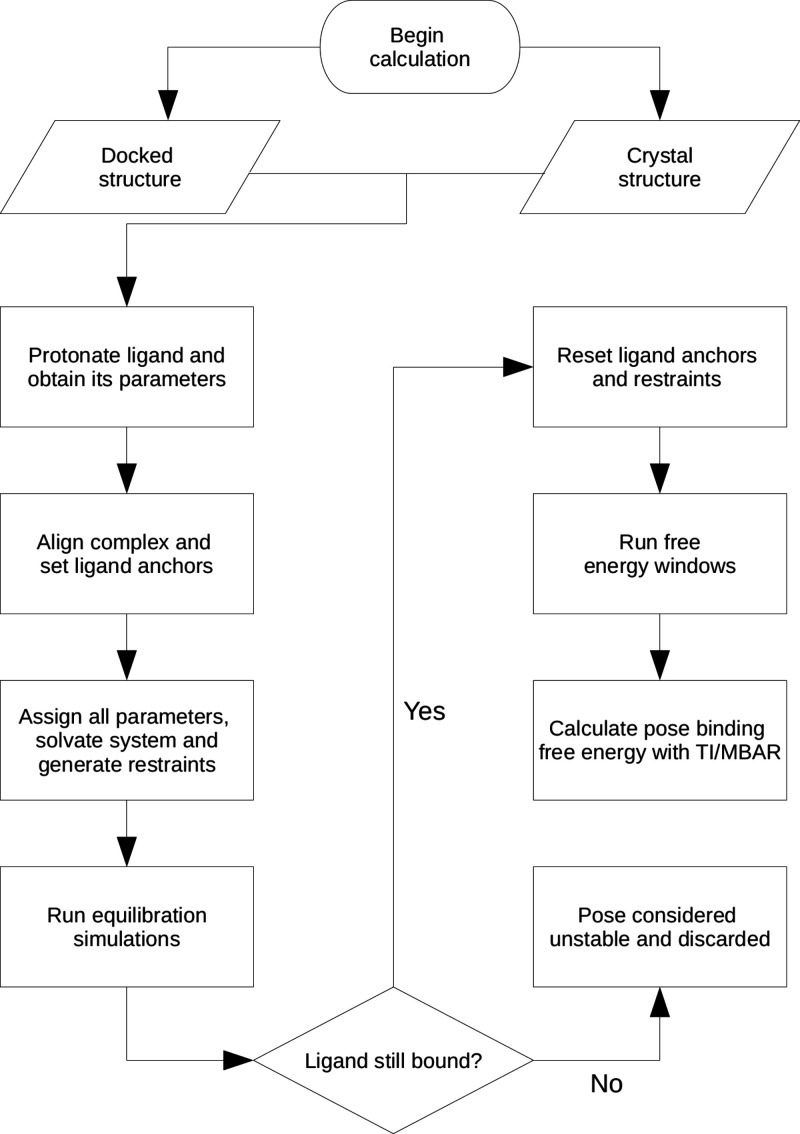
New workflow of the BAT.py software. See text for details.

- First, ABFE’s high computational cost when compared to scoring methods or even RBFE. In recent times, the widespread use of Graphics Processing Units (GPUs)^31−34^ has greatly increased the speed and scalability of MD simulations, making ABFE calculations considerably quicker and cheaper.
- Second, the human factor of building the systems and setting up the calculations manually. Recent tools such as BAT.py,^13^ the binding free energy estimator (BFEE),^35^ the CHARMM-GUI^36^ server and Schrodinger’s ABFEP^11^ have automated the steps of preparing, running and analyzing the necessary simulations, thus reducing or even eliminating the need of human intervention.
- Third, the need for the correct conformation of the ligand in the binding site in order to produce meaningful results. This challenge can be addressed by considering multiple different poses independently, which is incorporated in the BAT.py ABFE workflow (Figure 1). The pose with the lowest binding free energy would usually dominate the sum from eq 1, and thus be the one observed experimentally.
- Lastly, possible inaccuracies arising from conformational changes in the protein between its holo and apo states. The latter problem is discussed in detail in our previous work with Attach Pull Release (APR) calculations on bromodomains, in which such a transition is identified and its free energy contribution is rigorously computed.^21^ The BAT.py software also provides ways to address the issue of protein regions that have increased flexibility.

Since the release of the BAT.py 1.0 software (or BAT1) in 2020, and its associated article in 2021,^13^ there were important changes made to the code in order to make the calculations faster, easier, more rigorous and applicable to a wider variety of systems. Thus, in the present manuscript we introduce the BAT 2.x software, or BAT2, currently in its 2.3 version and available at https://github.com/GHeinzelmann/BAT.py. BAT2 provides several improvements over its predecessor, with the most important ones being.

- Support for the open-source OpenMM simulation software^37−39^ with OpenMMtools.^40^
- Relative restraints between the protein and the ligand, with a simpler procedure to add new receptors to the BAT2 workflow. Previously, both the protein and the ligand anchor atoms were restrained relative to fixed dummy atoms, which was harder to set up for larger proteins.
- The option of merging all attachments and releasing of restraints into two sets of simulation windows, which requires fewer simulations and makes the calculations cheaper.
- Applicability to proteins with multiple chains and in the presence of cobinders.
- Support for the TIP3PF^41^ and OPC^42^ water models, in addition to the ones already supported in BAT1.
- Automatic determination of the number of ions in all boxes for a chosen ion concentration.
- Use of the lovoalign software^43^ for protein structure alignment, replacing MUSTANG.^44^ The change was made because lovoalign can superimpose protein structures that contain multiple polypeptide chains.
- Choice of fixed solvation buffers in the three axes, or a fixed number of water molecules as with BAT1.
- Freedom to include ligands with hydrogens already added, as well as using pregenerated ligand parameters.
- Only two stages, equilibration and free energy calculations. This makes the calculations cheaper, since the preparation step is not needed in the BAT2 workflow (Section 3.1).

In the next sections we explain the theory and methods behind the modifications made, test them in terms of consistency with previous results, and use them on two sample systems. To help determine the feasibility of ABFE calculations on large libraries of ligands, we also explore the possibility of performing them with a small fraction of the simulation time used previously, assessing the effects of this reduction on their expected accuracy.

## 2 Theory

If we take into account all possible stable and nonoverlapping bound states of a given ligand in a given receptor’s binding site, *N*_*poses*_, the standard (or absolute) binding free energy of this molecule to a given receptor, Δ*G*°_*bind*_, can be determined using the equation:^13^
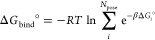
1where *i* indexes ligand poses, Δ*G*°_*bind*_ is the binding free energy computed for pose *i*, *R* is the gas constant, *T* is absolute temperature, and β^–1^ = *RT*.^45^ This expression assumes that the poses do not interconvert during their individual binding free energy calculations. Due to the exponential character of the term inside the sum, the lowest value of Δ*G*°_*i*_ will dominate the value of Δ*G*°_*bind*_, so we can consider these two quantities to be equivalent in most cases. The dissociation constant (*K*_*d*_) between the ligand (L) and the protein (P) is related to Δ*G*°_*bind*_ by the following expression:^26^
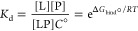
2where *C*° is the standard concentration of 1 M, [L], [P] and [LP] are the equilibrium concentrations of the respective species.

As commonly done for ABFE calculations, we will calculate the free energy of transferring the ligand from the binding site to bulk solvent (Δ*G*_*trans*_) in the presence of artificial restraints, in order to accelerate the convergence of the calculations. The final value of Δ*G*°_*bind*_ will then include, in addition to Δ*G*_*trans*_, the free energies of attaching and releasing the chosen restraints:
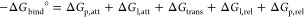
3

The values of Δ*G*_*p*,*att*_ and Δ*G*_*p*,*rel*_ represent the free energies of attaching and releasing restraints to the protein, respectively, and Δ*G*_*l*,*att*_ and Δ*G*_*l*,*rel*_ the same for the ligand. In Table 1 we list all the free energy components calculated by the BAT2 program, each identified by a letter. The way they are obtained, and how they correspond to each term from eq 3, will be explained in the next sections.

**Table 1:** Letter Codes for the Contributions to the Binding Free Energy Δ*G*_bind_°^a^

description	letter		system	method	term
attachment of protein conformational restraints	m	a	complex	MBAR	Δ*G*_p,att_
attachment of ligand conformational restraints		l	complex	MBAR	Δ*G*_l,conf,att_
attachment of ligand TR restraints		t	complex	MBAR	Δ*G*_l,TR,att_
ligand charge decoupling in site	e	e	complex^b^	MBAR/TI-GQ	Δ*G*_elec,bound_
ligand charge recoupling in bulk		f	bulk ligand^b^	MBAR/TI-GQ	–Δ*G*_elec,unbound_
ligand LJ decoupling in site	v	v	complex^b^	MBAR/TI-GQ	Δ*G*_LJ,bound_
ligand LJ recoupling in bulk		w	bulk ligand^b^	MBAR/TI-GQ	–Δ*G*_LJ,unbound_
release of ligand TR restraints	n	b	bulk ligand	Analytical	Δ*G*_l,TR,rel_
release of ligand conformational restraints		c	bulk ligand^b^	MBAR	Δ*G*_l,conf,rel_
release of protein conformational restraints		r	apo protein^b^	MBAR	Δ*G*_p,rel_

a The second column shows the merged m and n components for the attachment and release of restraints, as well as the electrostatic Δ*G*_elec_ (e) and Lennard-Jones (LJ) Δ*G*_LJ_ (v) components of the SDR procedure. TR stands for translational/rotational restraints, MBAR for Multistate Bennett Acceptance Ratio, and TI-GQ for Thermodynamic Integration with Gaussian Quadrature.

b For the SDR e and v components, and the merged n component, the complex (or apo protein) and the bulk ligand are placed far from each other in the same box.

## 3 Methods

### 3.1 BAT2 Workflow

The BAT2 automated workflow (Figure 1) encompasses all the steps needed to perform a full protein–ligand ABFE calculation, using either the double decoupling method (DDM)^26^ or the simultaneous decoupling and recoupling (SDR) method.^13,28^ BAT2 requires a few third-party programs such as OpenBabel,^46^ Visual Molecular Dynamics (VMD)^47^ and lovoalign, which are listed in the software’s main page and will be referred to throughout the manuscript. The simulations can be performed using either the *pmemd*.*cuda* software from AMBER,^48^ version 20 or later, or the OpenMM simulation engine with OpenMMtools, versions 7.7.0 or later for the former and 0.21.3 or later for the latter.

The main inputs to BAT2 are a protein–ligand pair, and an input file that has all the parameters needed for the calculation (Table 2). These include simulation times for each step, number of simulation windows for the free energy calculations, solvation options, as well as specific variables that have to be set up for a new receptor system. Also needed are a protein reference structure file and, depending on the system, molecule force-field parameters that were not generated automatically, as in the case of cobinders. The BAT2 User Guide provides further instructions on how to set up all the needed files, and can be found in the BAT2 distribution.

**Table 2:** Input Files Needed for the BAT.py Software

file description	file format	function	comments
docked receptor	PDB file	provides structure of protein/cobinders	can be replaced by a protein–ligand complex
docked poses	PDB files	docked ligand structures	not needed if using a protein–ligand complex
reference file	PDB file	contains the protein in the reference rotation	protein structure alignment performed with lovolign
BAT2 input file	text file	provides the BAT2 calculation parameters	includes the variables needed for a given protein system
additional parameters	.mol2 and .frcmod	simulation parameters for cobinders and ligand	the ligand parameters can also be generated automatically

As outlined in Figure 1, the workflow starts from either a receptor accompanied by a set of docked poses, or the structure of a docked complex such as a protein–ligand cocrystal structure. First, hydrogens are added to the ligand, and its force-field parameters to be used in the MD simulations are determined. The user can also maintain the ligand protonation and/or its parameters, in case they were already available before running BAT2. Next comes the alignment of the protein–ligand complex to a reference structure provided by the user, and determining the ligand anchor atoms that will be used in restraining the ligand relative to the protein. The complex is then solvated in water with a desired ion concentration, restraints are applied to the ligand relative to the protein, and equilibration simulations are performed in which these restraints are gradually released.

After the equilibration simulations are finished, the next step is the free energy step, which starts by checking if the ligand is still in the proposed binding site after equilibration (see section 3.2.1). If not, the initial docked pose is considered unstable and no free energy calculations are performed. If it is, the system is rebuilt in the equilibrated configuration but with a new set of ligand anchors and reference values for the restraints, and all the free energy simulations are carried out. Once the simulations are concluded, the analysis step will calculate the binding free energy of the ligand for that particular binding pose. The main BAT2 output is a file that contains the calculated free energy value for each component (Table 1), the sum of the components that make up binding free energy (eq 3), and the total simulation time needed for the calculation. BAT2 also outputs the equilibrated complex used as the starting point for the free energy step, which provides the reference coordinates used for the applied restraints. The binding free energies across different binding modes can then be evaluated to determine the correct one, and also compared to the values obtained for other molecules.

The main difference of the present workflow, when compared to BAT1, is the elimination of the preparation stage. This stage would carry out a procedure similar to steered molecular dynamics (SMD), generating initial states along the pulling coordinate used in the APR^21,24^ binding free energy method. This is not needed for BAT2, since the latter only applies the alchemical double decoupling (DDM)^26^ and the simultaneous decoupling and recoupling (SDR) methods^13,28^ for the ABFE calculations, the latter being suitable for ligands with net charge.

### 3.2 System Setup

Here there are several differences relative to the BAT1, so in the sections below we explain how BAT2 will use its input to set up the necessary systems and parameters for the calculations.

#### 3.2.1 Anchor Atom Selection

When applying BAT2 to a new protein system, three protein anchor atoms (P1, P2 and P3) have to be selected by the user, following a few rules to avoid gimbal-locking and effects on the internal degrees of freedom of the receptor. These rules include choosing backbone anchor atoms in regions that are typically rigid, such as alpha-helices, as well as avoiding short distances and angles between anchors that approximate 0 or 180°.

**Figure 2:**
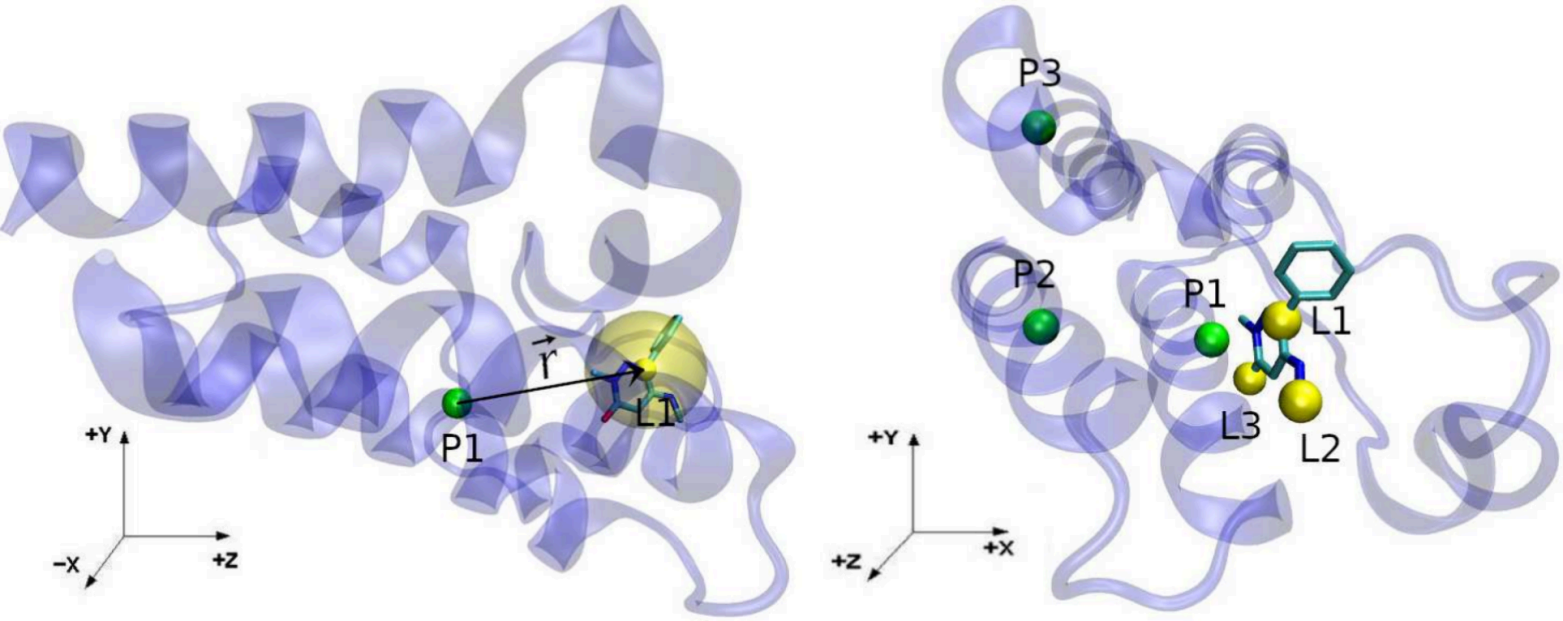
(Left) The P1 and L1 anchors, as the green and yellow solid spheres, and the r⃗ vector connecting the two. The transparent yellow region shows the L1 anchor spherical search region. (Right) All the protein and ligand anchors, as in the right of Figure 3 but from a different perspective.

Before the setup of the system for the equilibration and free energy stages, BAT needs to check if at least one atom of the ligand is inside the binding site, defined by a ligand search zone. This should be the case for the equilibration stage, since it starts from docked poses, but for the free energy stage the ligand might have left the pocket during the equilibration simulations (Figure 1). To try to find the ligand inside the pocket, BAT2 uses a spherical search zone with its center defined relative to the position of P1 using extrinsic Cartesian coordinates, not coordinates defined relative to the protein (Figure 2). For this reason, the protein needs to be in a predefined orientation, which is done by aligning the former to a reference structure using the program lovoalign. The reference structure, the coordinates of the center of the search zone and the search radius are all provided by the user. The BAT2 User Guide has detailed instructions on how to add a new protein system to the BAT2 workflow, with the help of visualization tools such as VMD or Chimera.^49^ Once that is concluded, any ligand that binds to the same binding site can be evaluated in a fully automated way without any human intervention.

The ligand anchors, called L1, L2 and L3, will be chosen automatically with the protein already in the reference orientation. The L1 anchor will be the ligand atom closest to the center of the selected search zone; if no ligand atoms are encountered inside the search zone at the start of the free energy stage, this means that the ligand has left the binding site during equilibration. In this case, the starting docked pose is considered to be unstable and no free energy calculation is performed (Figure 1). If L1 is found, the L2 anchor will be selected as the atom in which the P1-L1-L2 angle is closest to 90°, and the L1-L2 distance is within a specified range defined in the BAT2 input file. The choice of L3 follows the same procedure, but now using the L2-L3 distance and the L1-L2-L3 angle.

#### 3.2.2 Force Field Parameters, Solvation, and Ionization

If no information is provided by the user regarding the ligand protonation state and/or its simulation parameters, BAT2 will use the program Openbabel to add all hydrogens to the ligand molecule and estimate its net charge. The AM1-BCC charge model^50^ is then used on the protonated ligand to determine its partial charges, and versions 1 or 2 of the General AMBER Force Field (GAFF)^51,52^ for the LJ and bonded interactions. The user can also start with an already protonated ligand, choosing its net charge accordingly in the BAT2 input file, or generate the necessary ligand parameters separately and include them in the BAT2 workflow.

Regarding the protein, the protonation states of its titratable groups are predetermined from the associated residue templates. Several AMBER protein force-fields such as *ff14SB*(53) are available for use with BAT2, and can be selected by the user in the input file. The same goes for water models, with the ones currently supported by BAT2 being TIP3P,^54^ TIP3PF,^41^ TIP4PEw,^55^ SPC/E^56^ and OPC,^42^ with the associated Li/Merz cation and anion parameters for each.^57−60^ For the TIPEP, TIP4PEw and SPC/E water models, monovalent ions use the Joung and Cheatham ion parameters^61^ designed for that specific model. If there are other molecules or ion types in the system in addition to the protein, ligand, water and solvated ions, such as cobinders, the user needs to obtain the parameters for them separately before adding them to BAT2. In particular, the user should provide.mol2 and.frcmod files for each molecule, which can be generated using Antechamber by following the tutorial at https://ambermd.org/tutorials/basic/tutorial4b/index.php.

The Ambertools tleap software^48^ is used to solvate the system and add the ions to a concentration chosen by the user, or instead just add the counterions needed for neutralization. The user also specifies either the solvation buffers on the three Cartesian axes, or the buffers in two of the axes and the total number of water molecules that will be added to the system. These definitions will be used for all simulation boxes built for equilibration and free energy calculations, except the ones that have only the ligand in them. In the latter case, there will be specific solvation buffers for the small ligand box, with the ion concentration the same as the one chosen for the others.

### 3.3 Application and Release of Restraints

**Figure 3:**
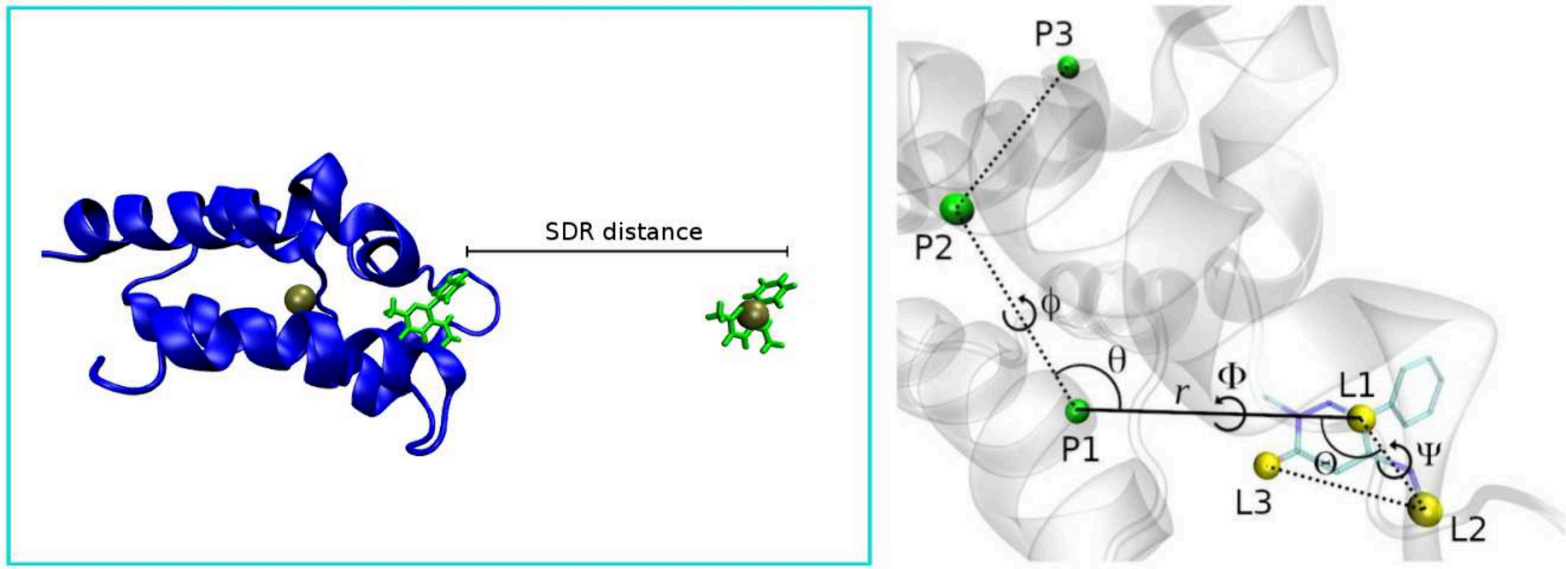
(Left) Depiction of the SDR box, with the protein in blue, the ligand in green, and the centers of mass of the protein backbone and bulk ligand as the golden spheres. The SDR distance is defined between the two ligands along the *z* axis and is chosen in the BAT2 input file. (Right) The TR restraints on the ligand relative to the protein, showing the protein anchors in green, the ligand anchors in yellow, and the six restrained degrees of freedom: the distance *r*; the θ and Θ angles; and the ϕ, Φ, and Ψ dihedrals.

We can separate the restraints applied to the protein and the ligand in three types: center of mass (COM) restraints, conformational restraints, and rigid-body translational/rotational (TR) restraints. The latter restrains both the position and orientation of the ligand relative to the protein using three anchor atoms in each molecule, with a total of six restrained degrees of freedom (Figure 3). These are commonly called the Boresch^23^ restraints, which are designed to keep the ligand in the binding site during the decoupling of the latter’s interactions with its environment. While there are RMSD-type restraint schemes such as the recent distance-from-bound-configuration (DBC),^62^ which apply all restraints in a single step, here we chose to separate the TR and conformational contributions, with the latter type being optional and not always necessary.

The COM restraints are used to maintain the location of the complex (or apo protein for consistency) inside the box, and also the position of the ligand in bulk solvent when the latter shares the simulation box with the first (left of Figure 3). These restraints are applied to either all backbone atoms of the protein or all non-hydrogen atoms of the bulk ligand, keeping the center of mass of the chosen atoms fixed throughout the simulation, but leaving each molecule free to rotate around its center of mass. No free energy calculations are performed for the COM restraints, since they only maintain the chosen reference frame and do not interfere with the internal degrees of freedom of a given species.

The conformational restraints on the protein are the same as previously described,^13^ so here we will go over them briefly. They are applied to one or more sections of the protein backbone, more specifically to the ϕ and ψ backbone dihedrals in this range, which may aid in convergence depending on the system. The free energies of attaching and releasing the protein backbone restraints correspond to the Δ*G*_*p*,*att*_ and Δ*G*_*p*,*rel*_ terms, respectively, and are calculated using a set of simulation windows with intermediate values of the applied spring constants. The first term is computed from simulations that have the ligand in the binding site, and the second has the protein in the apo state. The final attachment/releasing free energy differences are obtained from the respective simulations using the Multistate Bennett Acceptance Ratio (MBAR) method^63^ (Table 1). In the case of Δ*G*_*p*,*rel*_, this contribution is the same across poses if the protein restraint reference state is the same for all of them, and thus only has to be computed once.

The restraints applied to the ligand include both conformational and TR, so the Δ*G*_*l*,*att*_ and Δ*G*_*l*,*rel*_ terms from eq 3 will have two components each:
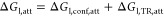
4
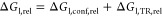
5

The Δ*G*_*l*,*conf*,*att*_ and Δ*G*_*l*,*conf*,*rel*_ terms correspond to the free energy of attaching and releasing the ligand conformational restraints. The same way as in our previous work,^13^ all dihedrals of the ligand that do not involve hydrogens are restrained with a spring constant specified by the user. The attachment of these restraints happens with the ligand in the binding site, and their release can happen either with the ligand in a separate box (DDM), or in the same box as the protein but at a distance in which they do not interact (SDR method). As with the protein conformational restraints, we use a number of simulation windows and obtain the final free energy difference using MBAR.

The ligand TR restraints on the BAT2 program are not relative to three fixed dummy atoms, as with BAT1, but instead they are defined relative to the protein. They involve one distance, two angles and three dihedrals between three protein anchors and the three ligand anchors as shown in Figure 3. The attachment of these restraints takes place when the ligand is in the binding site, using a series of simulation windows and the MBAR estimator. The release of the TR restraints to the standard concentration of 1 M is done analytically using eq 6:
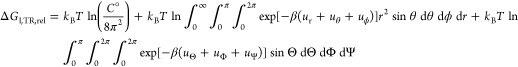
6, where *u* is the restraining potential applied to a given coordinate from Figure 3 (right) during the decoupling calculations.

#### 3.3.1 Merged Restraints

In BAT1, the attachment of the restraints in the bound state happens in sequence, so the ligand conformational free energy calculation has the protein conformational restraints already in place, and the ligand TR restraints free energy calculation has both the protein and ligand conformational restraints fully attached.^13^ New to BAT2 is the possibility of attaching and/or releasing all restraints using a single set of simulation windows with MBAR, which could reduce the computational cost of the calculations. We call these new components the merged restraint components, and they are identified by the **m** and **n** letters (Table 1).

The **m** component is the free energy of attaching all restraints to both the protein and the ligand in the bound state simultaneously, corresponding to the free energy term:
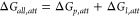
7, with the Δ*G*_*l*,*att*_ free energy contributions shown in eq 4. The **m** simulation boxes are identical to the **a**, **l**, and **t** ones, with the protein–ligand complex fully interacting in solution.

The **n** component computes the restraint releasing free energies for both the protein and the ligand, with the corresponding free energy difference:
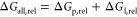
8, with the Δ*G*_*l*,*rel*_ term defined in eq 5. The two conformational contributions to Δ*G*_*all*,*rel*_, Δ*G*_*p*,*rel*_ for the protein and Δ*G*_*l*,*conf*,*rel*_ for the ligand, are calculated simultaneously using simulations that have the apo protein and the bulk solvent ligand in the same box but separated by COM restraints, as shown in Figure 3 but without the bound ligand. As done with the separated restraints, the remaining Δ*G*_*l*,*TR*,*rel*_ term is calculated analytically using eq 6.

### 3.4 Transfer of the ligand from binding site to bulk solvent

The transfer free energy in eq 3, Δ*G*_*trans*_, is defined as the free energy of decoupling the ligand interactions with its environment when it is in the bound state, and recoupling them back when the ligand is unbound in bulk solvent:
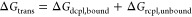
9In BAT2 there are two ways of performing the transformation of a protein-bound ligand to a bulk ligand: the double decoupling (DDM) and the simultaneous decoupling and recoupling (SDR) methods.^13^

In the case of DDM, four free energy calculations are performed, one for each of the following terms on the right-hand side of the expressions below:
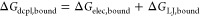
10
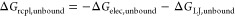
11Δ*G*_*elec*,*bound*_ is the free energy of decoupling all electrostatic interactions of the ligand with its environment in the bound state, and Δ*G*_*elec*,*unbound*_ the same for the ligand in bulk solvent. The Δ*G*_*LJ*,*bound*_ and Δ*G*_*LJ*,*unbound*_ terms follow the same definitions, but now for the Lennard-Jones interactions of a molecule that already has its charged interactions with the environment fully decoupled (also called a ”neutral” ligand). Following the letter identification from Table 1, the **e** and **v** DDM components will be performed on the solvated protein–ligand complex, and the **f** and **w** components in a smaller simulation box containing only the solvated ligand.

For the SDR method, only two free energy calculations are performed, each of them combining two components of the double decoupling method:
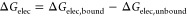
12
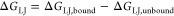
13

The SDR box has the protein–ligand complex and the bulk ligand in the same system (Figure 3), kept separated at a noninteracting distance by COM restraints applied separately to the receptor and the free ligand. The decoupling of the bound ligand and the recoupling of the bulk ligand take place simultaneously in the same system, both for the electrostatic (eq 12) and the Lennard-Jones component (eq 13). This approach keeps the net charge of the simulation box constant throughout the electrostatic leg of the calculation, avoiding numerical artifacts associated with charged ligands.^64−66^ The letter identifiers for the two SDR calculations are also **e** and **v**, the former for electrostatic and the latter for the LJ windows (Table 1). In contrast to the DD method, none of the SDR windows sample the true end-points of the calculations, which have the ligand fully coupled to the binding site and fully decoupled from bulk, and vice versa. Nonetheless, the same terms are being computed using the two approaches, which can be verified by comparing the three decoupling expressions for DDM (eqs 9, 10 and 11) and the two SDR expressions (eqs 12 and 13).

BAT2 can use either MBAR or Thermodynamic Integration with Gaussian Quadrature (TI-GQ)^21,48^ for the decoupling/recoupling calculations. OpenMM uses Hamiltonian Replica Exchange (HREX)^40^ when performing free energy calculations with MBAR, both for the attachment/release of restraints and for the transfer free energies, a feature that is not included in the AMBER calculations. The TI-GQ method requires the computation of the ensemble average of the derivative of the system potential energy *U* relative to the decoupling reaction coordinate λ, on a number of predetermined λ_*i*_ values, with the final value of the free energy difference given by
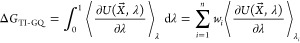
14with X⃗ the generalized coordinates for the position of the system particles. The expression on the right shows the integration using Gaussian quadrature for *n* windows, with its associated λ_*i*_ values and *w*_*i*_ Gaussian weights. There is an unique set of values for λ_*i*_ and *w*_*i*_ for each *n*, so the user only has to choose the value of the latter in the BAT2 input file.

In contrast with AMBER, it is not straightforward to obtain *∂U*/*∂λ* when using the OpenMM/OpenMMtools software. For this reason, we have developed a finite difference method to obtain this quantity when using the latter software, so that the TI-GQ method is available for both simulation packages. We make use of the following approximation, by considering ⟨*∂U*/*∂λ*⟩ to be constant along a small interval *δλ* (default value is *δλ* = 0.001):
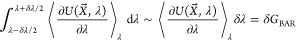
15

The value of *δG*_*BAR*_ above is obtained by using the OpenMM MBAR/HREX procedure on two decoupling windows located at λ_*i*_ – *δλ*/2 and λ_*i*_ + *δλ*/2, which is done for each point in which the derivative of the potential is to be calculated. The obtained derivatives are then plugged into the Gaussian Quadrature expression from eq 14 to obtain the desired value of Δ*G*_*TI*–*GQ*_.

### 3.5 Equilibration Stage

The equilibration simulations using AMBER start the same way as with the BAT1 program, with an initial minimization followed by 100 ps of heating from 10 K to the desired temperature using the Langevin thermostat.^67^ Then, a series of 15 ps simulations are performed to bring the system to 1 atm pressure using the Monte Carlo barostat.^68^ This procedure avoids possible AMBER crashes caused by excessive shrinking of the initial box. The OpenMM simulations added to BAT2 also start by performing an initial energy minimization on the solvated complex, after which the atom velocities are set to a random distribution that reflects the chosen temperature of the system. The simulation box is then coupled to a Langevin thermostat and a Monte Carlo barostat, in order to maintain the chosen temperature and a pressure of 1 atm.

The subsequent runs using both programs are carried out with constant temperature and pressure, with the parameters for the thermostat and barostat chosen in the BAT2 input file. A series of simulations slowly release TR and conformational restraints applied to the ligand, so that the surrounding protein has time to relax around the docked molecule. The protein conformational restraints, if chosen, can also be present at this stage. After the ligand restraints are removed, a (usually) longer simulation is performed, in which the ligand might find a nearby free energy minimum or leave the initial binding site. The magnitude of the restraints, and the simulation times for their release and for the unrestrained simulations, are all chosen by the user.

### 3.6 Free Energy Calculations

The free energy calculations are performed after equilibration, if the ligand has not left the binding site during the unrestrained simulations. The complex will be realigned to the reference structure, the ligand anchors and restraints will be redefined in order to reflect the equilibrated conformation, and for each chosen component of Table 1 (except Δ*G*_*l*,*TR*,*rel*_) a series of simulation windows will be created. They can be related to the attachment/release of restraints, or the transfer of the ligand from the binding site to bulk solvent. For each window, an initial equilibration is performed, followed by a production simulation in which data is collected.

Whereas for AMBER the user specifies a number of equilibration and production simulation steps for each window, in the case of OpenMM the user will select a number of equilibration/production HREX iterations and the number of MD steps for each. Other options for both programs can be selected in the BAT2 input file, such as the number of windows and the intermediate lambda values.

Once all simulations from the free energy step are concluded, BAT2 will compute the free energy contribution from each component (Table 1) used in the calculation, and the final binding free energy between the protein and the ligand for that particular binding mode. The uncertainties will be computed from block data analysis as done with the BAT1 version,^13^ with the number of data blocks chosen by the user.

### 3.7 Protein–Ligand Test Systems

In order to exemplify the use of the BAT2 program, we will apply it to two protein systems. The first is the second bromodomain of the Bromodomain-contaning protein 4, or BRD4(2), bound to a unique fragment that was used as a starting point for a series of new binders,^69^ the same system used in our previous study on the BAT1 program.^13^ As in the latter, we will perform calculations with the ligand from the 5uf0 crystal structure docked to the 5uez receptor, as well as the on the 5uf0 crystal structure itself.

The second one is the human HIV-1 protease protein, also bound to a small molecule, with PDB ID 5ivq.^70^ This system is suitable to demonstrate some of the new features of BAT2, such as support for proteins with multiple chains, inclusion of protonated residues and the presence of cobinders. Here we will also perform ABFE calculations on 5 docked poses and the original 5ivq cocrystal structure.

### 3.8 Computational Details

For all simulations we use rectangular periodic boxes, with a cutoff value of 9.0 Å and long-range electrostatics calculated using Particle Mesh Ewald (PME).^71^ AMBER uses the SHAKE^72^ and SETTLE^73^ algorithms to keep rigid all bonds involving hydrogens, and OpenMM additionally uses the CCMA algorithm^74^ for the same purpose. All simulations in this study use hydrogen mass repartitioning (HMR),^75^ and a 4.0 fs time step. In the HMR procedure, performed using the Ambertools *parmed* program, the mass of each hydrogen is multiplied by a factor of 3 and this enhanced hydrogen mass is subtracted from the atom to which the hydrogen is bonded. Soft-core potentials are applied to the Lennard-Jones interactions during the ligand LJ decoupling calculations, using the soft-core parameters default values for both AMBER and OpenMM. All other parameters used for the calculations, such as lambda values and simulation times, can be found in the BAT2 input files included in the Supporting Information (SI).

## 4 Results and Discussion

In this section we test and validate the new methods included in BAT2 software, such as new restraint schemes and support for OpenMM, by comparing its results to the ones from the original BAT release on BRD4(2).^13^ We also use use this same system to test calculations with very short time scales, aiming to reduce their computational cost. Finally, we apply the BAT2 workflow to a HIV-1 protease system, and evaluate the calculations in terms of accuracy, robustness, and computational cost.

### 4.1 BRD4(2)

For this protein, we perform all the ABFE calculations starting from the five equilibrated poses and cocrystal structure from our previous study,^13^ so they have the same starting points and restraint reference states, allowing the results to be directly compared.

#### 4.1.1 SDR and Merged Restraints

We perform two types of calculations for each system: a double decoupling procedure with each restraint component calculated separately, and the SDR procedure using the merged restraint scheme described in Methods. The first one corresponds to all the letters in the third column of Table 1, and we will call it ”split”. The second corresponds to the letters in the second column of the same table, and we will call it ”merged”. We use both the AMBER and the OpenMM simulation engines in each case, and summarize the results in Table 3. Detailed results, with the separate values obtained for each free energy component, can be found in the Supporting Information Also included in the SI are all the needed files to reproduce the results shown here in an automated way using the BAT2 software.

**Table 3:** Binding Free Energy (Δ*G*_bind_) Results (in kcal/mol) Using Different Methods for the Five Equilibrated Docked Poses and the Equilibrated 5uf0 Cocrystal Structure, with the Associated Uncertainties in Parentheses^a^

	crystal	pose 1	pose 2	pose 3	pose 4	pose 5
RMSD (Å)	1.30	5.26	0.45	5.33	4.23	0.74
BAT 1.0	–6.1 (0.6)	–2.5 (0.9)	–6.7 (0.6)	–2.6 (0.8)	–1.5 (0.6)	–6.5 (0.8)
split AMBER	–7.0 (1.5)	–2.7 (1.2)	–5.9 (1.5)	–1.7 (1.3)	–1.5 (1.9)	–7.5 (1.2)
merged AMBER	–6.8 (1.5)	–3.5 (1.0)	–5.7 (0.8)	–3.3 (1.3)	–2.8 (1.5)	–7.3 (1.2)
split OpMM	–6.6 (0.8)	–2.9 (0.8)	–5.9 (0.9)	–1.3 (1.1)	–1.3 (1.0)	–6.8 (0.8)
merged OpMM	–6.2 (1.1)	–2.4 (1.4)	–6.1 (0.7)	–3.7 (0.8)	–1.5 (1.2)	–7.2 (0.8)

a Also shown are the ligand RMSDs of the initial equilibrated states relative to the initial 5uf0 structure. The BAT1 calculations used the “split” procedure with the AMBER software.

Table 3 shows the calculated binding free energies, using BAT1 and BAT2, for six equilibrated states obtained from ref.,^13^ five starting from docked poses and one from the 5uf0 crystal structure. Table 3 also displays the ligand structural root-mean-square deviation (RMSD) relative to the 5uf0 crystal structure, for each of the six starting states. The RMSD value is used to quantify the similatity between a ligand binding mode, generated by simulations or docking, to the one determined experimentally.

For all starting structures, there is good agreement between the results that use the AMBER and OpenMM softwares, the split and merged procedures, and generally between BAT1 and BAT2. The original BAT 1.0 article uses a total of 1.16 μs of simulations for each calculation using the split scheme, while here we use 148 ns for the same procedure, using either AMBER or OpenMM. The merged method uses a total of 100.8 ns for both softwares, bringing a more than 10-fold reduction in the simulation time needed for a single calculation when compared to the BAT1 calculations.^13^ Still, the differences between the two methods remain inside the computed uncertainties (around 1 kcal/mol), which is the case for the other protocols as well. We do notice a slight increase in the uncertainties when the shorter times are used, even though the results seem to be robust across the five different calculation types. To exemplify the convergence of the results shown in Table 3, in Figures S1 and S2 from the SI we show the free energy value of each component as a function of time for pose 2, when using the merged method with AMBER. As mentioned above, these calculations are much shorter than the ones from BAT 1.0, but Figures S1 and S2 show that they still display good convergence.

It is also important to compare the SDR and merged components to their split counterparts, in order to demonstrate that the merged free energy terms are being computed correctly. If the initial and reference states are the same, the free energy value of the merged **m** component should be the sum of the **a**, **l** and **t** free energies from the split method, and the merged **n** component should be the sum of the split **b**, **c** and **r** components. Also, the **e** component of the SDR method should be the sum of the **e** and **f** components of the DD method, and the same goes for the SDR **v** component when compared to the DD **v** and **w** free energies. This comparison is shown in Table 4 for the OpenMM software, with the same comparison for AMBER included in the SI. We observe good agreement for all merged components, with no discrepancies over 1.1 kcal/mol and most of them inside the uncertainty values. The merged method uses less simulation time and is suitable for ligands with net charge, so it might be preferable over the split one in most cases.

**Table 4:** Comparison between the Free Energies (in kcal/mol) Using the Merged and Split Schemes, for Each of the Merged Restraints and SDR Components

	attach restraints		electrostatic		Lennard-Jones		release restraints	
system	merged **m**	split **a, l, t**	SDR **e**	DD **e, f**	SDR **v**	DD **v, w**	merged **n**	split **b, c, r**
pose 1	28.2 (0.6)	29.3 (0.4)	–0.7 (0.3)	–0.5 (0.2)	12.2 (1.0)	12.5 (0.6)	–37.3 (0.6)	–38.4 (0.5)
pose 2	26.5 (0.3)	26.5 (0.3)	3.2 (0.2)	3.5 (0.3)	12.4 (0.6)	12.1 (0.6)	–36.1 (0.2)	–36.2 (0.5)
pose 3	28.2 (0.4)	27.8 (0.4)	–1.2 (0.3)	–1.4 (0.3)	12.1 (0.4)	11.5 (0.9)	–35.4 (0.3)	–36.5 (0.4)
pose 4	31.2 (0.6)	31.6 (0.4)	0.3 (0.3)	0.5 (0.3)	7.7 (0.9)	6.6 (0.8)	–37.6 (0.5)	–37.4 (0.3)
pose 5	26.3 (0.3)	26.5 (0.3)	3.6 (0.1)	2.8 (0.4)	12.7 (0.4)	13.5 (0.5)	–35.3 (0.6)	–35.9 (0.3)

### 4.2 Reducing Simulation Time

Computational cost has always been a barrier to apply ABFE calculations on a high-throughput scale, even with the significant gains in performance obtained with the use of GPUs. Thus, one of our main goals with BAT2 is to significantly reduce the time necessary for a single ABFE calculation.

With that in mind, here we will perform two types of short calculations on the BRD4(2) system: one of them using a total of 17.4 ns of simulations per pose, and another using a total of 20.4 ns. We will call the first one the short **tevb** calculation, which as the name suggests uses only these four components, with the **e** and **v** components using the SDR method. No conformational restraints are applied to the ligand or the protein, so their corresponding free energy contributions are not present. The second type we will call the short m*evbc calculation, which also uses SDR and applies conformational restraints to the ligand only. The m* component applies the ligand TR and conformational restraints using a single set of windows, with the ligand conformational restraints being released in a small box (**c** component) and the ligand TR release analytically (**b** component).

We have performed six ABFE calculation replicas, all using OpenMM, for each equilibrated pose from Tables 3 and 4, and the results are shown in Tables 5 and 6. Even though we have drastically reduced the time necessary for a single ABFE calculation, the results are consistent with the longer calculations, and also similar between independent replicas. Most importantly, the calculations are still able to identify the correct binding modes, with poses that have an RMSD under 2.0 Å (2 and 5, Table 3) always showing a higher affinity when compared to the other ones. There is also reasonable agreement between the binding free energy of these two poses with the experimental value of −5.2 kcal/mol,^69^ even though there is a more pronounced overestimation of the affinities when compared to the longer BAT 1.0 calculations.

**Table 5:** Calculated Binding Free Energies for the Six Replicas of Each Pose, Using the Short tevb Procedure^a^

tevb calculation (17.4 ns)					
replica	pose 1	pose 2	pose 3	pose 4	pose 5
1	–2.5 (1.8)	–6.9 (1.2)	0.0 (0.9)	–2.0 (0.7)	–8.6 (1.2)
2	–0.5 (0.8)	–7.2 (0.6)	–1.0 (0.9)	–0.2 (0.7)	–8.7 (0.9)
3	–3.1 (1.4)	–4.5 (0.6)	–4.0 (1.0)	–1.7 (1.8)	–8.4 (1.2)
4	–1.2 (1.0)	–8.6 (1.0)	–2.2 (1.0)	–0.6 (1.2)	–7.4 (1.1)
5	–3.4 (0.8)	–8.1 (1.2)	–2.1 (0.6)	–2.7 (1.5)	–6.9 (0.9)
6	–1.2 (0.9)	–6.0 (0.9)	–3.1 (1.1)	–3.3 (1.4)	–6.5 (0.9)
average	–2.0 (1.1)	–6.9 (1.4)	–2.1 (1.3)	–1.8 (1.1)	–7.7 (0.9)
long	–2.4 (1.4)	–6.1 (0.7)	–3.7 (0.8)	–1.5 (1.2)	–7.2 (0.8)

a The uncertainties for the replicas are computed through the usual block data analysis, and the uncertainty of the averages are the standard deviation across replicas. For comparison, the last row shows the results for the longer calculations using 100.8 ns (last row of Table 3).

**Table 6:** Same as Table 5, but Using the Short m*evbc Procedure for the Six Replicas^a^

m*evbc calculation (20.4 ns)					
replica	pose 1	pose 2	pose 3	pose 4	pose 5
1	–3.3 (1.1)	–7.2 (1.6)	–2.2 (0.9)	–1.1 (1.1)	–8.1 (0.8)
2	–3.6 (1.4)	–7.4 (1.1)	–3.2 (1.2)	–3.3 (1.1)	–6.2 (1.3)
3	–1.8 (1.2)	–7.4 (0.8)	–2.7 (1.1)	0.5 (2.0)	–7.2 (0.6)
4	–3.4 (1.3)	–7.6 (1.4)	–3.5 (1.3)	–1.3 (1.4)	–8.9 (1.0)
5	–3.2 (1.7)	–7.0 (1.6)	–3.0 (0.8)	–0.7 (1.0)	–6.9 (1.4)
6	–1.8 (1.2)	–7.4 (1.7)	–2.6 (1.5)	–2.4 (2.1)	–6.8 (1.3)
average	–2.9 (0.8)	–7.3 (0.2)	–2.9 (0.4)	–1.4 (1.2)	–7.4 (0.9)
long	–2.4 (1.4)	–6.1 (0.7)	–3.7 (0.8)	–1.5 (1.2)	–7.2 (0.8)

a Here also the last row shows the results for the longer calculations using 100.8 ns.

The short calculations from this section retain surprising accuracy when compared the ones performed using much longer time scales, reducing the computational cost by a factor of more than 50 when compared to our previous BAT1 results. Even though the **tevb** approach uses slightly less simulation time, the ligand does not have its conformation restrained during the application of the TR restraints and the decoupling/recoupling steps. BAT uses three anchor atoms on the protein and three on the ligand to define the relative position and rotation of the ligand as a whole. This means that anchor atoms placed in highly flexible regions of the ligand could potentially overestimate the magnitude of the TR attachment free energy contribution.^23^ Since the ligand anchor atoms are chosen automatically based on geometrical criteria only, an approach that keeps the ligand rigid such as m*evbc is usually recommended. The only additional simulations in that case, when compared to a **tevb** calculation, are the release of the ligand conformational restraints in a small box (**c** component), which has a relatively low computational cost.

It is also important to put these short calculations in terms of computational effort. Using the BAT2 workflow with the OpenMM software, a single NVIDIA GTX 1070 GPU can perform a short calculation for a single pose from Tables 5 and 6 in 2.6 and 2.75 h, respectively. Since the calculations can be run doing trivial parallelization across windows, components, poses and ligands, it is possible for a server with a few hundred GPUs to perform several thousands of calculations in the time frame of a few weeks. Newer graphics cards such as the RTX 30 and 40 series can significantly reduce the time needed for a single calculation, thus also increasing the number of ligands that can be tested in a given time.

### 4.3 HIV-1 Protease

**Figure 4:**
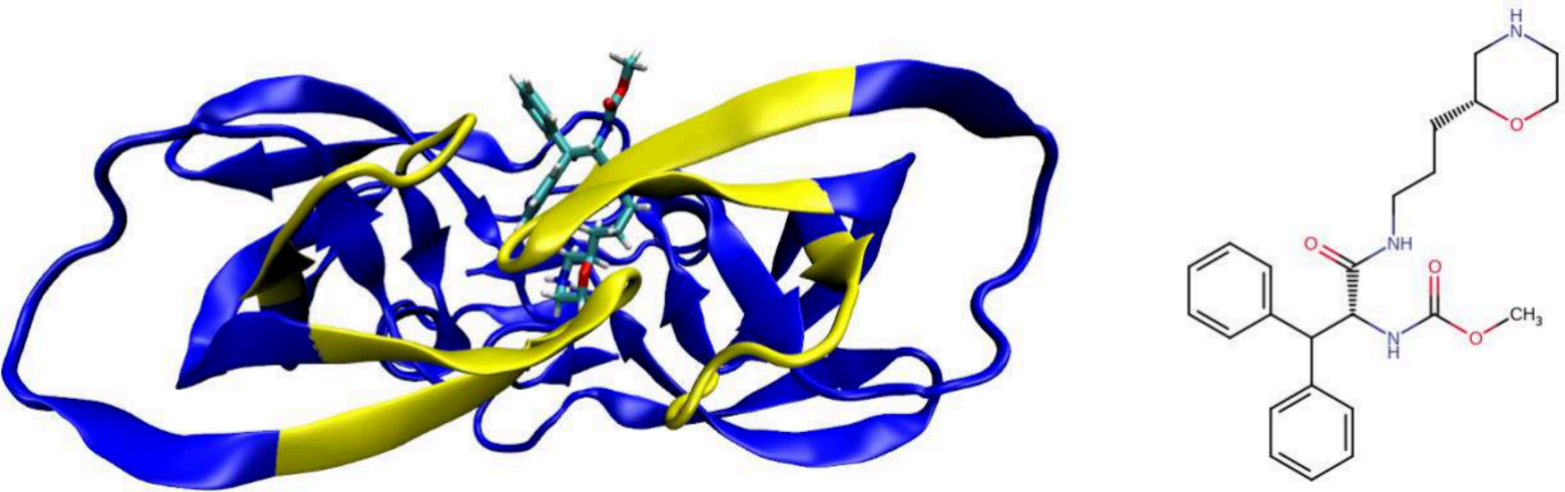
(Left) The 5ivq structure of the HIV-1 protease, with the restrained backbone regions in yellow. (Right) The ligand from this structure, where we include a +1 net charge on the morpholine ring.

To illustrate some of the new features of BAT.py, we also apply our workflow to the HIV-1 protease system with PDB ID 5ivq (Figure 4). Binding free energy calculations are performed after equilibration on five self-docked poses using the Autodock Vina^76^ software, and also on the equilibrated 5ivq crystal structure. We choose OpenMM for all calculations performed in this section, choosing two different sets of free energy components.

The first set has the protein backbone restraints applied to four noncontiguous sections of the protein backbone (Figure 4), which we call **mevn**, or simply ”merged” as in section 4.1.1. The second does not include protein conformational restraints, and we name m*evbc as in section 4.2. Both use a total of 100.8 ns of simulations per ABFE calculation, but m*evbc is slightly cheaper, due to the smaller size of the **c** component simulation box when compared to **n**. Complete results and all input files, including the initial structures and the BAT2 parameters used for both approaches, are included in the SI.

The results are shown in Table 7. For both methods, the docked pose that is closest to the crystal structure, which is pose 3, has the highest affinity. The binding free energy values obtained for this pose are also near the values obtained for the known crystal structure, even though they are slightly lower, but still within the uncertainties (added in quadrature). The differences between the **mevn** and m*evbc methods for all poses, even though larger that in the case of the BRD4(2) bromodomain, are also inside the computed uncertainties. Thus, we believe that the discrepancies are mostly due to the natural fluctuations associated with ABFE calculations.

**Table 7:** Binding Free Energies (in kcal/mol) for the Five Docked and Equilibrated Poses, As Well As for the Equilibrated 5ivq Cocrystal Structure^a^

	crystal	pose 1	pose 2	pose 3	pose 4	pose 5
RMSD (Å)	0.35	5.48	9.01	1.65	5.64	8.91
mevn	–7.3 (1.5)	2.5 (2.4)	–1.5 (1.9)	–9.6 (2.8)	–0.4 (2.4)	0.1 (2.0)
m*evbc	–7.2 (1.4)	4.2 (1.7)	0.1 (1.1)	–9.7 (1.9)	–2.7 (1.6)	–3.0 (1.3)

a Also shown are the ligand RMSDs of the equilibrated states relative to the initial 5ivq structure.

The experimental data for this ligand reports 11% inhibition of HIV-1 protease, at a ligand concentration of 1 μM and a protein concentration of 20 pM.^70^ This puts the affinity roughly between −7 and −8 kcal/mol, and thus near the values calculated using BAT2. Thus, for this example we were able to correctly identify the correct docked pose, even though it was not the one with the best Vina score, and also produce binding free energy values comparable to experiments.

## 5 Conclusions

We have presented here the BAT2 software, currently in its 2.3 version, explaining the theory behind it and testing it on two sample systems. On the first, good agreement is observed between calculations performed using AMBER or OpenMM, and also between different choices for the free energy components that make up the total binding free energy. We also show a significant reduction in the computational cost of ABFE calculations, making it now possible to test hundreds of thousands of ligands on a reasonable time. The second system illustrates some of the new BAT2 features, that now make it applicable to virtually any protein–ligand system. Here the results are also consistent with the available experimental data, with BAT2 being able to find the experimental binding pose and to correctly estimate the binding free energy.

When compared to its earlier version BAT1, BAT2 is more broadly applicable, easier to set up for new systems, cheaper to run due to the merged windows scheme, and compatible with the free and open-source OpenMM simulation engine. BAT.py itself is an open-source software, freely available for download at the GitHub platform.

## Supporting Information Available

Figures S1 and S2, a quick installation guide for the BAT dependencies, additional tables with detailed results, and all input files needed to reproduce the results from this work (PDF)
