# Correction to “BAT2: an Open-Source Tool for Flexible, Automated, and Low Cost Absolute Binding Free Energy Calculations”

**DOI:** 10.1021/acs.jctc.4c01153

**Published:** 2024-09-17

**Authors:** Germano Heinzelmann, David J. Huggins, Michael K. Gilson

The current published version did not include a few supporting files, namely, two tables containing detailed results of the calculations (Table S1 and Table S2) and also the input files needed to reproduce the paper results. The two tables are inside the tables.zip file, and the input files are inside input-files.zip, both uploaded as Supporting Information in this addition submission.

## Supporting Information Available

input-files.zip (ZIP)
tables.zip (ZIP)

