# Supplementary material for: BAT2: an Open-Source Tool for Flexible, Automated, and Low Cost Absolute Binding Free Energy Calculations
Source: J Chem Theory Comput. 2024 Aug 1;20(15):6518–30. doi: 10.1021/acs.jctc.4c00205 (PMC11325538; doi:10.1021/acs.jctc.4c00205)
Supplement: Supplementary file 1 — ct4c00205_si_001.pdf [file ct4c00205_si_001.pdf]

Supporting Information

BAT2: An open-source tool for flexible, automated and low cost absolute binding free energy calculations

Germano Heinzelmann [*,†], David Huggins [‡,¶], and Michael Gilson[§]

[†]Departamento de Física, Universidade Federal de Santa Catarina, Florianópolis, Brasil

[‡]Department of Physiology and Biophysics, Weill Cornell Medical College of Cornell University, New York, New York 10065, United States

[¶] Sanders Tri-Institutional Therapeutics Discovery Institute, 1230 York Avenue, Box 122, New York, New York 10065, United States

[§] Skaggs School of Pharmacy and Pharmaceutical Sciences, University of California, San Diego, USA

Figure S1: Free energy values as a function of simulation time for the merged attach (black) and merged release (red) free energy components for pose 2 of the BRD4(2) bromodomain system. Shown are the absolute values for better comparison.

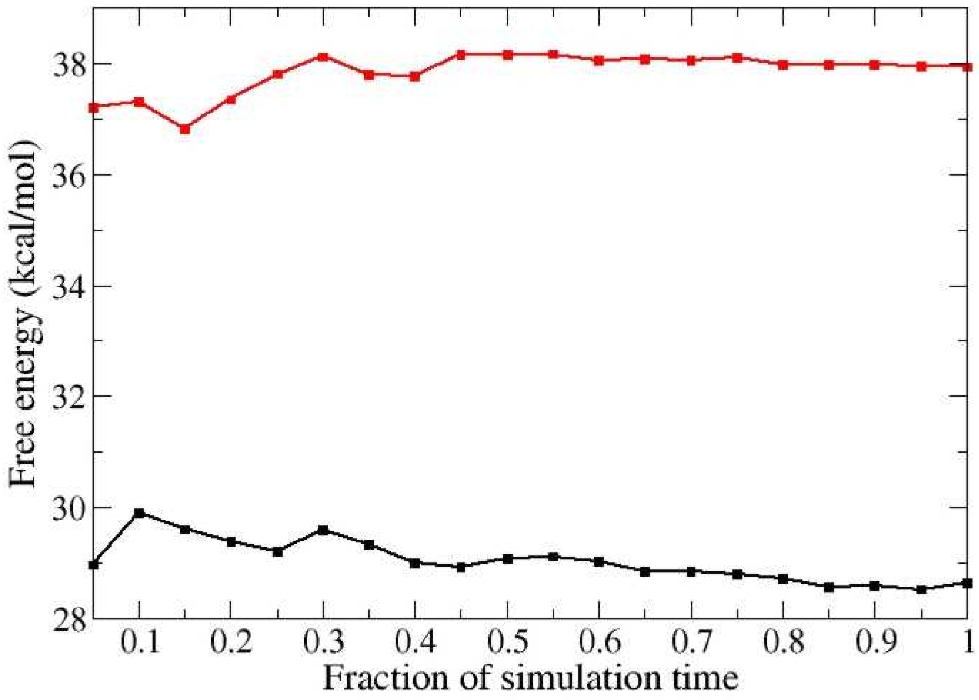

Figure S2: The same as Fig. S1, but for the electrostatic free energy component (black), Lennard-Jones (red) and the final binding free energy (green) of the same calculation (pose 2 from BRD4(2). The simulation time for the full calculation (green line) is 100.8 ns.

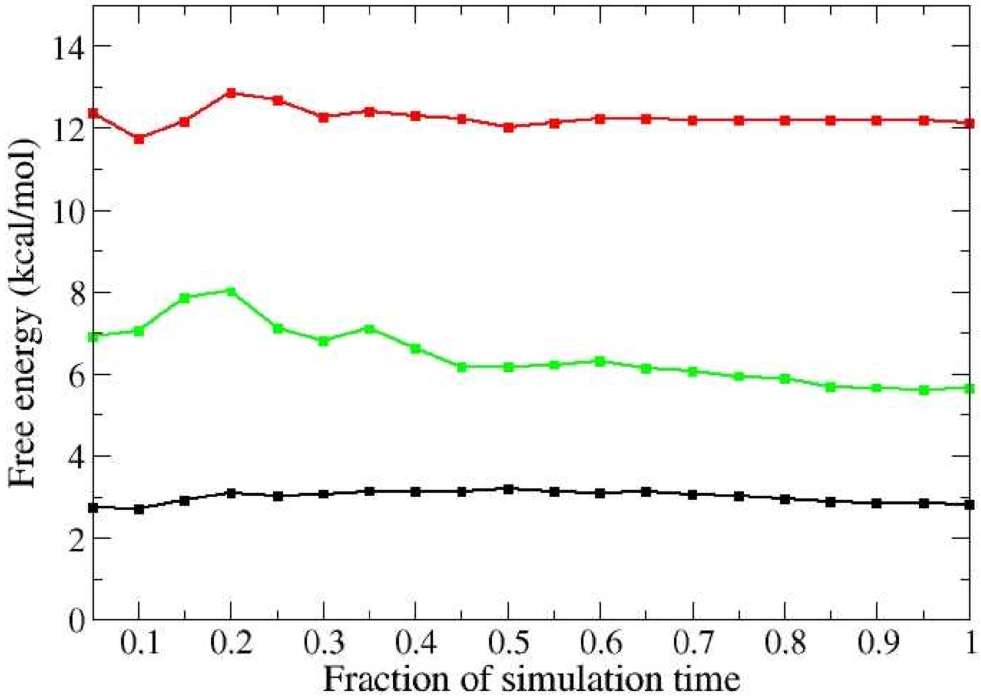

Quick installation and tutorial Guide

This document explains how to install BAT.py and its needed dependencies, and running the BAT tutorial, on a Linux machine with an OS such as Ubuntu. We make use here of the Anaconda package manager, which makes it easier to install some of the needed software.

Installation

To make BAT.py operational on your system, follow the steps below. If you already have an Anaconda installation and the needed environment, you can skip to step 2.

1. Install Anaconda: First download and install the Anaconda package manager, from the page:

https://www.anaconda.com/download

Follow the instructions on this page, and download the Linux version of Anaconda to your folder of choice, such as your home directory. For most local machines the Linux x86 version should be chosen.

Install Anaconda from the file you downloaded, typing the command below and following the instructions:

```
you@yourmachine:~$ bash Anaconda3-2024.02-1-Linux-x86_64.sh
```

After the installation, close all your terminal windows, and open a new window. Create a new Anaconda environment and activate it, or activate a created environment of your choice. Instructions on how to create and activate Anaconda environments can be found here https://conda.io/projects/conda/en/latest/user-guide/tasks/manage-environments.html#.

You might need to install gfortran and/or lapack for some of the programs to work properly, which can be done through Anaconda as well. Already inside your chosen Anaconda environment, type the following commands:

```
(environment)you@yourmachine:~$ conda install conda-forge::lapack
```

```
(environment)you@yourmachine:$ conda install conda-forge::gfortran
```

2. Install Ambertools: Already inside your chosen Anaconda environment, you will now install some of the software needed for BAT.py, starting with Ambertools. Type in your command line:

```
(environment)you@yourmachine:~$ conda install -c conda-forge
ambertools=23
```

You can also install Ambertools in other ways, with instructions here https://ambermd.org/GetAmber.php.

3. Install VMD 1.9.3: To install VMD in your Anaconda environment, type:

```
(environment)you@yourmachine:~$ conda install conda-forge::vmd
```

4. Install OpenMM with OpenMMtools: If you want to use OpenMM with OpenMMtools for your simulations (more details in the BAT tutorial), install both of them in your Anaconda environment. Do that by typing these two commands and following the instructions:

```
(environment)you@yourmachine:~$ conda install conda-forge::openmm
```

```
(environment)you@yourmachine:~$ conda install conda-forge::openmmtools
```

5. Install Openbabel 2.4.1: Even though Anaconda provides the 3.1 version, here we want to use the 2.4.1 version instead. Thus, we do not recommend installing the Anaconda version of Openbabel, because it will then execute the 3.1 version.

To install the 2.4.1 version, download the source code zip file from the GitHub page https://github.com/openbabel/openbabel/releases/tag/openbabel-2-4-1 . Unzip it in your folder of choice, typing:

```
you@yourmachine:~$ unzip openbabel-openbabel-2-4-1.zip
```

Follow the (Basic) installation instructions in the INSTALL file, inside the just created ./openbabel-openbabel-2-4-1 folder.

5. Install lovoalign: To install lovoalign, go to their software page at https://www.ime.unicamp.br/~martinez/lovoalign/home.html. Go to the “Download” item of the side menu, and follow the installation instructions.

6. Install *pmemd.cuda:* If you wish to use AMBER’s *pmemd.cuda* for your simulations, instead of OpenMM with OpenMMtools, you will need to install this software as well. This can be done by downloading Amber24 from the https://ambermd.org/GetAmber.php page, and following the installation instructions.

7. Download BAT.py: If you have not yet done so, download the BAT.py distribution from the GitHub page https://github.com/Gheinzelmann/BAT.py. Unzip the downloaded file at your folder of choice, typing:

```
(environment)you@yourmachine:~$ unzip BAT.py-master.zip
```

You will now see a ./BAT.py-master folder, contaning the program and the needed files for the tutorial. The tutorial will be performed inside the ./BAT.py-master/BAT folder.

8. Run the tutorial: Now activate your Anaconda environment, which should have all the needed programs installed. Also, check if all the other needed programs are also installed, and in your path. Perform the tutorial as explained in the BAT GitHub page, or following the simplified command-oriented instructions below.

Performing the tutorial using OpenMM

Once you have everything installed as explained above, run the following command on your command line, inside the ./BAT folder:

```
(environment)you@yourmachine:~$ python BAT.py -i input-sdr-openmm.in -s equil
```

Now, to run the equilibration simulations using OpenMM, go the pose folders that were created inside the ./BAT/equil folder, and on each run the command:

```
(environment)you@yourmachine:~$ source run-local.bash
```

You can also use the PBS and SLURMM files provided, whose templates are inside the ./BAT/run_files folder. Once all the equilibration simulations are *finished*, go back to the ./BAT folder and type:

```
(environment)you@yourmachine:~$ python BAT.py -i input-sdr-openmm.in -s fe
```

Now go inside each pose folder in the ./BAT/fe directory, and copy the run-op-express.bash file from the ./BAT/run_files folder to the current one. This bash script is adjusted for SLURMM, edit it if running the simulations locally or using PBS. Now run the script:

```
(environment)you@yourmachine:~$ source run-op-express.bash
```

This will run several simulations at the same time, so make sure your local environment or server has its GPUs properly set. Once all the the simulations are finished, inside the ./BAT folder type:

```
(environment)you@yourmachine:~$ python BAT.py -i input-sdr-openmm.in -s analysis
```

Now inside each pose folder in the ./BAT/fe/ directory there should be a Results folder. There, the user can find all the calculated free energies for the blocks and for the whole run.
